# The invisible scars: a critical perspective on the social-**behavioral** impact of emotional abuse in Ugandan students

**DOI:** 10.3389/fpsyg.2026.1902178

**Published:** 2026-09-23

**Authors:** Florence Patricia Nakacwa

**Affiliations:** Department of Educational Psychology, Nkumba University, Entebbe, Uganda

**Keywords:** emotional abuse, psychosocial wellbeing, qualitative research, reflexive thematic analysis, school counseling, school-based violence, trauma-informed education, Uganda

## Abstract

**Introduction:**

School-based emotional abuse remains an under-recognized form of childhood adversity within many educational settings, despite growing evidence of its potential influence on students' psychosocial wellbeing. This qualitative study explored how students and school personnel in six primary and secondary schools in Entebbe Municipality, Wakiso District, Uganda, described and interpreted experiences of emotional abuse and their perceived social and behavioral consequences.

**Methods:**

A descriptive qualitative design was employed. Forty-six participants were purposively recruited, comprising 28 students aged 12–18 years, 12 teachers, and six school counselors. Data were generated through 22 semi-structured individual interviews, four student focus group discussions involving six participants each, and 10 supplementary open-ended questionnaires. Data were analyzed inductively using Braun and Clarke's (2006, 2019) six-phase Reflexive Thematic Analysis. Data collection and preliminary analysis proceeded concurrently, and saturation was judged to have been reached when successive interviews and focus groups generated no new codes, sub-categories, or distinct contextual variations.

**Results:**

Participants described emotional abuse through corporal punishment accompanied by humiliation, public shaming, peer bullying and exclusion, and perceived teacher neglect. These experiences were associated in participants' accounts with anxiety, emotional withdrawal, reduced classroom participation, school avoidance, interpersonal difficulties, and anger or other externalizing responses. Recurring contextual patterns concerned gender, boarding vs. day-school contexts, and institutional capacity, while supportive teachers, peer relationships, and guidance and counseling services were identified as protective resources.

**Discussion:**

The findings position school-based emotional abuse as an important educational and child-protection concern. Strengthening positive discipline, trauma-informed teacher education, accessible psychosocial support, and school-family collaboration may help promote emotionally safer learning environments. The findings are context-specific and offer qualitative, transferable insight rather than statistical generalization.

## 1 Introduction

### 1.1 Background and scope

Childhood is a foundational period for emotional regulation, social competence, identity development, and emerging perceptions of self-worth. When school experiences include repeated humiliation, rejection, chronic criticism, psychological intimidation, or relational exclusion, students may experience distress that extends beyond the immediate interaction. Contemporary child-protection evidence increasingly recognizes psychological violence and bullying as important forms of harm affecting children's wellbeing and participation in education (Gaffney et al., 2021; World Health Organization, 2024; World Health Organization the United Nations Educational, 2024; Scientific and Cultural Organization, 2024). Although physical violence has historically received greater attention in child-protection research and policy, emotional and psychological maltreatment within educational environments can be less visible precisely because some practices are embedded in routine discipline, peer interaction, or institutional culture.

In Uganda, national evidence documents substantial exposure of children to violence, including violence occurring in and around educational settings Uganda Bureau of Statistics, 2018; Wandera et al., 2024). Research and intervention evidence from Uganda has also demonstrated the importance of school-level approaches to reducing violence (Devries et al., 2021; Knight et al., 2021). However, emotional abuse is not always examined as a distinct experiential phenomenon. Public ridicule, severe verbal discipline, peer ostracism, and chronic shaming may be normalized as discipline or peer interaction, potentially obscuring their psychological significance (Ssenyonga et al., 2022). Qualitative attention to how students and school personnel interpret these experiences is therefore important for understanding how emotional harm becomes visible, how it is expressed, and what forms of support students perceive as protective.

### 1.2 Theoretical framework

This study used three complementary theoretical perspectives as interpretive lenses rather than as pre-specified causal models. Bronfenbrenner and Morris's (2006) Bioecological Model helped situate students' accounts within proximal school interactions and wider institutional and sociocultural contexts. Erikson (1963) Psychosocial Development Theory provided a developmental lens for interpreting accounts involving competence, self-worth, belonging, and identity. Resilience Theory (Masten, 2018) was used selectively to interpret participant-described protective resources, particularly supportive teacher–student relationships, peer solidarity, and access to counseling. Consistent with the qualitative design, these perspectives were used after and alongside the empirical analysis to illuminate participants' accounts rather than to test hypotheses or establish causal pathways.

### 1.3 Research gap and objectives

Despite growing recognition of school violence in East Africa, important empirical gaps remain. First, intervention research in Uganda has demonstrated the value of school-wide violence-prevention approaches, but emotional abuse is often discussed alongside broader categories of violence rather than examined through students' and school personnel's own descriptions. Second, relatively little qualitative attention has been given to how school context—particularly residential vs. non-residential arrangements—may shape students' experiences and access to support. Third, the ways in which distress becomes visible or remains hidden across gendered and institutional contexts warrant further contextual exploration.

Accordingly, this study sought to: (i) characterize the predominant forms of school-based emotional abuse described by students and school personnel; (ii) examine how participants described the associated social, emotional, and behavioral responses; and (iii) explore contextual influences, particularly gender, school type, and institutional capacity, in participants' accounts of emotional abuse and its consequences.

## 2 Methods

### 2.1 Research design

The study adopted a descriptive qualitative design (Sandelowski, 2000; Colorafi and Evans, 2016; Doyle et al., 2020). This approach was selected because the study sought a rich, straightforward account of a sensitive and under-explored educational phenomenon across three stakeholder groups. The study did not adopt a phenomenological methodology. Data were analyzed using Braun and Clarke (2006, 2019) Reflexive Thematic Analysis, allowing patterned meanings to be identified across participant accounts while retaining contextual detail.

### 2.2 Study setting and school selection

The study was conducted in Entebbe Municipality, Wakiso District, Central Uganda. Six schools were purposively selected to maximize structural variation across three explicit criteria: ownership (three government-aided and three private schools), educational level (three primary schools covering P.5–P.7 and three secondary schools covering S.1–S.4), and school type (three day and three boarding schools). Formal administrative authorization was obtained from the relevant municipal education authorities and the participating head teachers before recruitment commenced.

### 2.3 Participants and sampling strategy

Forty-six participants were recruited through purposive sampling: 28 students aged 12–18 years (15 female and 13 male), 12 teachers (seven female and five male; 3–14 years of teaching experience), and six school counselors, one from each participating school. Students were initially approached with the assistance of school guidance counselors and, where appropriate, were invited to share study information with peers who might voluntarily wish to participate. This counselor-facilitated student-to-peer referral process was used cautiously because of the sensitivity of the topic. Teachers were eligible if they had at least 2 years of classroom experience, while counselors were eligible because of their direct responsibility for student welfare, guidance, or psychosocial support. Student participation required willingness to discuss school-related experiences in a manner that did not require disclosure of identifiable information.

### 2.4 Data collection procedures and instrumentation

Data collection occurred over 3 months. 22 semi-structured individual interviews were conducted with all 12 teachers, all six counselors, and four students who preferred a private setting. Individual interviews lasted approximately 25–35 min and were conducted in private rooms at the participating schools. Four student focus group discussions were conducted, with six students per group (24 students in total), and lasted approximately 45–60 min. Group participation was used only where the setting was considered appropriate for the sensitive topic; students who preferred privacy were offered individual interviews. 10 supplementary open-ended questionnaires were completed by a subset of participants (six teachers and four counselors) who wished to elaborate in writing after their oral interviews. Field notes documented contextual observations, non-verbal cues, and reflections made during and immediately after data collection. All audio-recorded interviews and focus groups were transcribed verbatim before analysis.

### 2.5 Sampling adequacy and data saturation

Data collection and preliminary analysis proceeded concurrently. Sampling adequacy was assessed using an operational understanding of saturation (Saunders et al., 2018), defined here as the point at which successive interviews and focus group discussions generated no new codes, sub-categories, or distinct contextual variations relevant to the study questions. On this basis, saturation was judged to have been reached after the 22 individual interviews and four focus group discussions, after which recruitment was concluded. The 10 written questionnaires were supplementary elaborations from participants already represented in the interview sample and were not treated as a separate basis for determining saturation.

### 2.6 Data analysis framework

Analysis followed Braun and Clarke (2006, 2019) six-phase Reflexive Thematic Analysis. First, the researcher familiarized herself with the dataset through repeated reading of transcripts alongside audio files and field notes. Second, initial codes were generated inductively through line-by-line coding. Third, related codes were clustered into candidate sub-categories and themes. Fourth, candidate themes were reviewed against the coded extracts and the full dataset for coherence and distinctiveness. Fifth, themes were defined and named. Sixth, the final analytic account was produced, linking descriptive findings to the study questions while reserving broader theoretical interpretation for the Discussion. Coding trees, thematic definitions, and analytical memos were reviewed through peer debriefing with two experienced qualitative researchers. The researcher also maintained a reflexive journal documenting positionality, interpretive decisions, and changes to the coding framework.

### 2.7 Methodological rigor and trustworthiness

Trustworthiness was considered in relation to credibility, dependability, confirmability, and transferability (Lincoln and Guba, 1985). Credibility was supported through source triangulation across students, teachers, and counselors and method triangulation across interviews, focus groups, written responses, and field notes. Ten purposively selected participants reviewed summaries of the emerging findings and confirmed that the interpretations were recognizable to their accounts; no substantive changes were requested. Dependability and confirmability were supported through an audit trail comprising recordings, verbatim transcripts, coding trees, analytical memos, and a reflexive journal. Transferability was supported through detailed description of the study setting, school characteristics, participant groups, and analytic procedures, enabling readers to judge relevance to comparable contexts.

### 2.8 Ethical considerations and safeguards for minors

The study was reviewed and approved by the Nkumba University Research Ethics Committee. Ethical approval was granted without a formal reference number being assigned. The study was conducted in accordance with the Declaration of Helsinki (World Medical Association, 2013) and applicable Ugandan national research ethics guidance (Uganda National Council for Science Technology, 2021). Written informed consent was obtained from adult participants. For participants younger than 18 years, written informed consent was obtained from their parents or legal guardians, together with written assent from the minors, before participation. Participation was voluntary, and participants were informed of their right to decline participation or withdraw from the study without penalty.

Participant and school identities were protected through pseudonymization and de-identification of transcripts and the removal of personally identifying information from the reported findings. Given the potentially distressing nature of the experiences explored, appropriate safeguards were put in place to minimise psychological distress. A referral pathway to school counselling and appropriate community-based psychosocial support services was established and made available during data collection for participants who required additional support. The demographic characteristics of the participants are presented in Table 1.

**Table 1:** Qualitative analytical progression matrix: initial codes to subthemes and final themes.

Initial open codes (Phase 2)	Subtheme (Phase 3)	Final theme (Phases 4–5)
Public caning with verbal degradation; degrading comments during punishment	3.1.1 Corporal punishment as emotional harm	Theme 1: Forms of school-based emotional abuse
Public reading of low test scores; derogatory labels; humiliating tasks	3.1.2 Public shaming and humiliation	Theme 1: Forms of school-based emotional abuse
Dormitory isolation; rumors; peer exclusion; verbal taunting; intimidation	3.1.3 Peer bullying and relational aggression	Theme 1: Forms of school-based emotional abuse
Selective attention to high performers; ignoring struggling students; dismissal of distress	3.1.4 Teacher neglect and emotional exclusion	Theme 1: Forms of school-based emotional abuse
Fear of teachers; headaches/stomach discomfort before classes; attendance avoidance	3.2.1 Anxiety and school avoidance	Theme 2: Social, emotional and behavioral responses
Silence; eating alone; loss of interest; reduced peer interaction	3.2.2 Emotional withdrawal and sadness	Theme 2: Social, emotional and behavioral responses
Anger; fighting; confrontation; resistance to authority	3.2.3 Anger and externalizing responses	Theme 2: Social, emotional and behavioral responses
Reduced trust; isolation; dependence on a single trusted peer	3.2.4 Social withdrawal and difficulties in peer relationships	Theme 2: Social, emotional and behavioral responses
Relational exclusion and self-silencing; observable anger or defiance	3.3.1 Gender dynamics and expressions of distress	Theme 3: Contextual influences
Prolonged peer contact; limited access to family support; residential exposure	3.3.2 School type: boarding vs. day	Theme 3: Contextual influences
Large classes; high teacher workloads; limited counseling capacity	3.3.3 Teacher-to-student ratios and institutional capacity	Theme 3: Contextual influences
Teachers who listen without judging; individual adult support	3.4.1 Supportive teacher–student relationships	Theme 4: Protective factors
Trusted friendships; mutual reassurance; peer solidarity	3.4.2 Peer solidarity and mutual support	Theme 4: Protective factors
Confidential counseling; referral pathways; psychosocial support	3.4.3 Guidance and counseling services	Theme 4: Protective factors
Public exposure during punishment	3.1.1 Corporal punishment as emotional harm	Theme 1: Forms of school-based emotional abuse
Fear of social judgement after mistakes	3.1.2 Public shaming and humiliation	Theme 1: Forms of school-based emotional abuse
Hyper-vigilance around peers	3.2.4 Social withdrawal and difficulties in peer relationships	Theme 2: Social, emotional and behavioral responses
Early identification through counseling	3.4.3 Guidance and counseling services	Theme 4: Protective factors

The audit matrix below shows the progression from 18 representative open codes to the 14 subthemes reported in the Results and four final themes. The matrix is a transparency aid rather than a frequency table; it does not imply quantitative weighting of codes or themes.

## 3 Results

Reflexive thematic analysis generated four interconnected themes: (1) forms of school-based emotional abuse; (2) social, emotional, and behavioral responses; (3) contextual influences; and (4) protective factors. The analysis progressed from 18 initial open codes to 14 reported subthemes and four final themes. The subthemes were supported by anonymized verbatim quotations from students, teachers, and school counselors. Findings are presented descriptively, while broader theoretical interpretation is reserved for the Discussion.

### 3.1 Forms of school-based emotional abuse

#### 3.1.1 Corporal punishment as emotional harm

Participants described corporal punishment as emotionally harmful when physical discipline was accompanied by public exposure, degrading language, or attacks on dignity. The concern in these accounts was not physical pain alone but the humiliation and loss of status experienced in front of peers.

“*The physical pain goes away after some time. The real damage is how it is done to you in front of everyone. You feel ashamed long after the punishment is finished.”* — 15-year-old male boarding student.

A senior teacher similarly observed: “*Sometimes the child is not only corrected but exposed and degraded to show control.”* These accounts indicate that public humiliation and degradation were central to how participants interpreted the emotional harm associated with some disciplinary practices.

#### 3.1.2 Public shaming and humiliation

Public shaming was repeatedly described through public disclosure of poor academic performance, derogatory labels, and humiliating tasks. Participants associated these practices with a loss of confidence and reluctance to become visible in class.

“*After being laughed at in front of everyone, you stop raising your hand even when you know the answer.”* — Secondary student.

Participants linked repeated embarrassment with self-silencing and reduced classroom participation. The accounts suggest that academic mistakes could become socially threatening when correction was delivered through public humiliation.

#### 3.1.3 Peer bullying and relational aggression

Participants described peer-directed ridicule, exclusion, intimidation, rumors, and relational aggression across school settings. Boarding participants emphasized the difficulty of distancing themselves from harmful peer interactions.

“*You cannot easily run away from bullying because you see the same people in the dormitory, classroom, and during meals.”* — Boarding student.

The accounts suggest that prolonged peer proximity could make exclusion and intimidation more difficult to escape, contributing to fear, loneliness, and reduced belonging.

#### 3.1.4 Teacher neglect and emotional exclusion

Teacher neglect was described as being ignored, noticed mainly when making mistakes, or receiving little emotional encouragement. Participants also connected this experience with large classes and limited teacher capacity.

“*Some students feel invisible. Teachers only notice you when you have done something wrong.”* — Male student, day school.

Participants experienced limited recognition as emotional exclusion, particularly when students perceived that academic performance determined the amount of attention or support they received.

### 3.2 Social, emotional, and behavioral responses

#### 3.2.1 Anxiety and school avoidance

Participants described fear of particular teachers, reluctance to attend classes, and physical complaints associated with anticipated disciplinary or humiliating interactions.

“*Some students complain of severe headaches or stomach pain, especially on days when they know they have certain teachers. When you look closely, the problem is not a physical sickness itself; it is deep fear of what may happen to them in that classroom.”* — School counselor, primary day school.

Teachers and counselors associated some attendance difficulties and somatic complaints with specific classroom experiences. The findings therefore describe perceived links in participants' accounts rather than clinical diagnoses.

#### 3.2.2 Emotional withdrawal and sadness

Emotional withdrawal was described as a quiet response involving reduced communication, eating alone, loss of interest, and limited peer interaction.

“*I stopped talking to people because I felt nobody really understood what I was going through. I started eating alone and keeping quiet because being silent was much easier than trying to explain how hurt I felt inside.”* — 16-year-old female boarding student.

A senior counselor added:

“*The learners who withdraw emotionally are often the hardest to identify and support because they are not causing trouble in class. They become almost invisible in a large school because their pain is quiet.”*

These accounts show how internalizing distress may remain less visible in routine school systems.

#### 3.2.3 Anger and externalizing responses

Participants also described anger, fighting, verbal confrontation, and resistance to authority. Teachers noted that such behaviors were often treated first as disciplinary problems.

“*When a teacher humiliates you in front of the whole class, you feel completely trapped. If you cry or show that you are hurt, fellow students laugh at you. Sometimes the only way you can respond to defend your dignity is by getting angry.”* — 15-year-old male secondary student.

A secondary teacher reflected:

“*As teachers, we often punish the aggressive behavior immediately without stopping to ask what triggered it. A learner may appear stubborn or rebellious, but there is usually a painful story behind that reaction.”*

The accounts suggest that some visible behavior was interpreted by participants as a response to humiliation or frustration.

#### 3.2.4 Social withdrawal and difficulties in peer relationships

Participants described reduced trust, hyper-vigilance, isolation, and strong attachment to a single trusted peer as responses to emotionally harmful experiences.

“*Some students attach themselves strongly to just one peer because they no longer trust the broader group, while others isolate themselves completely. Both patterns are protective walls they build after being emotionally hurt.”* — Counselor, secondary boarding school.

These accounts indicate that students used different interpersonal strategies to manage perceived social risk. Participants described these responses as individualized coping patterns rather than a single uniform outcome.

### 3.3 Contextual influences: gender, school type, and institutional capacity

#### 3.3.1 Gender dynamics and expressions of distress

Gender-related patterns concerned the visibility and expression of distress rather than differences in severity. Female participants more frequently described relational exclusion, public embarrassment, and self-silencing, while teachers more often described some male students' distress through observable anger or defiance.

“*After being embarrassed in front of others, you become afraid of speaking because you think people will laugh at you again.”* — 15-year-old female secondary student.

A teacher observed:

“*Sometimes boys show their pain through anger. What appears to teachers as bad behavior or rebellion may actually be a protective reaction to what they have experienced.”*

The pattern suggests differences in how distress may become visible to school staff, not a deterministic difference between genders.

#### 3.3.2 School type: boarding vs. day schools

Boarding participants described prolonged exposure to peers and school personnel and fewer opportunities to return to family environments when distressed. The analysis does not support a claim that boarding schools inherently have more emotional abuse.

“*When something happens at school, you cannot easily escape it because you meet the same people in class, in the dormitory, and during meals.”* — 16-year-old male boarding student.

Another boarding student explained:

“*You learn to hide when you are hurt because showing weakness can make others target you more in the dormitories.”*

Participants also identified supportive teachers, counselors, and peers as protective resources within boarding settings.

#### 3.3.3 Teacher-to-student ratios and institutional capacity

Participants identified large classes, teacher workloads, and limited counseling resources as conditions that could constrain individualized attention and early identification of emotional distress.

“*When the class is very large, some learners become invisible. You notice the students who are disruptive, but the quiet ones who are struggling emotionally can easily be missed.”* — Primary school teacher.

A male day-school student similarly stated: “*Some students feel like they do not exist to the school. Teachers only notice you when you have done something wrong or made a mistake.”* These accounts position teacher neglect partly within structural capacity constraints.

### 3.4 Protective factors within the school environment

#### 3.4.1 Supportive teacher–student relationships

Participants described trusted teachers who listened, showed empathy, and responded without judgement as important sources of emotional safety.

“*Sometimes one teacher who listens without judging can make a student feel that someone finally understands what they are going through. That single safe connection can change how a learner views the entire school.”* — School counselor, secondary day school.

The account illustrates how one supportive adult relationship could provide recognition and reduce isolation, although participants noted that workloads could limit sustained individual support.

#### 3.4.2 Peer solidarity and mutual support

Trusted friendships were described as informal sources of reassurance and belonging, particularly when students felt misunderstood or criticized.

“*When I talked to my friends, I realized I was not the only one feeling that way or being criticized. Knowing they had my back helped me keep coming to school.”* — 15-year-old female secondary student.

Participants described small friendship networks as spaces where vulnerability could be expressed without fear of judgement. Such networks were viewed as protective, although they did not replace formal support.

#### 3.4.3 Guidance and counseling services

Students and school personnel described counseling as a confidential space for discussing emotional concerns and interpersonal difficulties. At the same time, access and capacity varied across schools.

“*When a student finds a counselor who listens and keeps the conversation safe, they can begin to talk about things they have hidden for a long time.”* — School counselor.

Participants noted constraints including high student-to-counselor ratios, limited private counseling space, and competing administrative duties. Counseling was therefore valued as a protective resource whose availability and effectiveness depended on institutional capacity. The main themes and subthemes emerging from the qualitative analysis are presented in Table 2.

**Table 2:** Summary of qualitative thematic findings across school contexts.

Form of school-based emotional abuse	Associated social, emotional and behavioral expressions	Contextual influences identified	Summary of participant accounts
Corporal punishment with humiliating commentary	Anxiety, fear, school avoidance	Boarding context; disciplinary authority structures	Participants emphasized psychological harm associated with public exposure and degradation rather than physical pain alone.
Public shaming and humiliation	Reduced participation, self-silencing, lowered confidence	Large classes; limited individualized support	Students described avoiding classroom participation after repeated public embarrassment.
Peer bullying and relational aggression	Social isolation, reduced trust, hyper-vigilance, emotional distress	Boarding residential density; peer interaction patterns	Participants described exclusion, ridicule, and intimidation as compromising belonging and psychological safety.
Teacher neglect and emotional exclusion	Diminished self-worth, disengagement, reduced help-seeking	High teacher workloads; structural class capacity	Students described feeling “invisible” when recognition and emotional support were limited.

## 4 Discussion

### 4.1 Interpretation of the findings

Participants' accounts described school-based emotional abuse through public humiliation, harsh verbal discipline, peer exclusion and bullying, and perceived teacher detachment or neglect. They associated these experiences with anxiety, withdrawal, reduced classroom participation, school avoidance, and difficulties in peer relationships, while some described anger or other externalizing responses. These findings are broadly consistent with evidence linking violence and psychological maltreatment in educational contexts with poorer psychosocial and social outcomes (Devries et al., 2021; Gaffney et al., 2021; World Health Organization the United Nations Educational, 2024; Scientific and Cultural Organization, 2024). Importantly, the present study adds qualitative detail about how students and school personnel make sense of these experiences in a Ugandan school context.

### 4.2 Theoretical interpretation

The theoretical interpretation begins with participants' accounts rather than treating theory as a causal model. Boarding students' descriptions of prolonged peer contact and limited access to family support illustrate the relevance of Bronfenbrenner and Morris's (2006) Bioecological Model: students' immediate school interactions are embedded within wider family, institutional, and sociocultural contexts. Similarly, students' accounts of public humiliation, reduced confidence, and self-silencing can be interpreted through Erikson's (1963) concepts of competence, inferiority, and identity development. Finally, participants' emphasis on supportive teachers, peers, and counseling provides a basis for using Resilience Theory (Masten, 2018) to understand protective resources. These theories illuminate the accounts but do not establish causal relationships.

### 4.3 Contextual influences

Gender and school-type patterns were treated as contextual patterns within this participant group. Teachers and counselors more often described some male students' distress through observable anger or defiance, while female participants more often described withdrawal and self-silencing. This pattern is compatible with literature on gendered expressions of distress (Knight et al., 2021; Wandera et al., 2024), but it should not be interpreted as a fixed or universal distinction. Boarding participants described prolonged exposure to peer and school interactions and fewer opportunities for family contact. These accounts suggest that residential context may shape the experience and coping environment, but they do not establish that boarding schools have greater levels of abuse than day schools.

### 4.4 Implications for policy and practice

The findings support recognition of emotional abuse as part of the broader school-violence and child-protection agenda. Practical responses suggested by participants' accounts include strengthening positive discipline, preparing teachers to recognize less visible forms of distress, expanding accessible guidance and counseling, developing trauma-informed school practices, and strengthening school–family collaboration. These implications are offered as practice and policy directions arising from a context-specific qualitative study rather than as tested intervention effects.

### 4.5 Study limitations

The study has several limitations. First, purposive sampling across six schools in one municipality limits statistical generalization. Second, the sensitive nature of the topic may have shaped disclosure, including the possibility that some participants withheld experiences or emphasized socially acceptable accounts. Third, the analysis was conducted by the primary researcher, although peer debriefing, member checking, triangulation, an audit trail, and reflexive journaling were used to strengthen credibility and confirmability. Fourth, the study included different stakeholder groups and school contexts, which improved contextual breadth but also means that findings should be interpreted as patterns across accounts rather than as estimates of prevalence. Future studies using larger multi-site samples, longitudinal designs, and mixed methods could examine the distribution and persistence of the patterns identified here.

## 5 Conclusion

This study provides qualitative evidence that students and school personnel in six schools in Entebbe Municipality described school-based emotional abuse through teacher humiliation and harsh verbal discipline, corporal punishment accompanied by degrading treatment, peer exclusion and bullying, and perceived teacher neglect. Participants associated these experiences with anxiety, emotional withdrawal, reduced classroom participation, school avoidance, interpersonal difficulties, and anger or other externalizing responses. Recurring contextual patterns concerned gender, boarding vs. day-school contexts, and institutional capacity, while supportive teachers, peer relationships, and guidance and counseling services were consistently described as protective resources.

These findings are context-specific and are not intended to establish causal relationships or statistical generalizations. Nevertheless, they reinforce the importance of recognizing emotional abuse within school-based violence and child-protection responses. Positive discipline, trauma-informed teacher preparation, accessible psychosocial support, and stronger school–family partnerships are practical areas for attention. Further research across diverse Ugandan educational settings is needed to test, extend, and contextualize the patterns identified in this study.

## Data Availability

The raw data supporting the conclusions of this article will be made available by the author, without undue reservation.
